# Eat, pray, love: disordered eating in religious and non-religious men and women

**DOI:** 10.1186/s40337-022-00721-8

**Published:** 2022-12-20

**Authors:** Danie A. Beaulieu, Lisa A. Best

**Affiliations:** Department of Psychology, University of New Brunswick, 100 Tucker Park Road, Saint John, NB E2L 4L5 Canada

**Keywords:** Disordered eating, Appearance-related pressures and internalizations, Religiosity, Gender

## Abstract

**Background:**

Despite the lack of research examining the relation between religiosity and disordered eating, at various points during the year, religious practices requiring changes in eating habits are typical. Few studies have identified how aspects of religiosity are associated with disordered eating attitudes and behaviours. Thus, we explored the interconnectedness of religiosity and gender on disordered eating attitudes and behaviours.

**Methods:**

In total, 749 religious and non-religious participants completed online questionnaires assessing components of disordered eating and associated appearance-related pressures and internalizations (pressure from family, peers, and media, and internalization of the thin and muscular ideals).

**Results:**

Among the 317 participants who identified as religious, 12.30% reported that their religious practice required a change in their eating habits, and 10.68% reported that they changed their eating habits for both religious purposes and as a method of weight loss/control. Overall, religious participants who indicated changing their eating habits for religious purposes experienced greater disordered eating and appearance-related pressures than theists who reported no change in their diet and non-religious respondents. Further, there was a significant interaction between gender and religiosity across the disordered eating variables. Results indicated that, compared to males who were not religious, those who were religious had higher scores on scales measuring disordered eating. Religious and non-religious women scored similarly on scales measuring other aspects of disordered eating (including Purging, Restricting, and Binge Eating). Further, compared to non-religious men, religious men, reported greater pressure from their family and peers; there was no difference in women.

**Conclusions:**

Future research should further explore gender differences across types and specific aspects of religiosity such as motivation to practice.

## Plain Language Summary

Studies have identified benefits and risks of aspects of religiosity in disordered eating. Our purpose was to explore the role of gender and religiosity in disordered eating. We found that, in males, religiosity was associated with increases in all aspects of disordered eating as well as increased pressure to maintain the thin ideal. Among religious and non-religious females, there were fewer differences in disordered eating attitudes and behaviours. Religious and non-religious females reported similar pressures to attain the thin ideal. Thus, future research should further examine the underlying factors of religiosity related to disordered eating in men and women.

## Background information

In the literature, dieting has been defined as deliberately restricting calorie intake for a prolonged period of time to decrease body fat [1]. In some studies, dieting has encompassed both healthy and unhealthy weight loss behaviours in which dieting was measured with the single question “How often have you gone on a diet during the last year? By “diet” we mean changing the way you eat so you can lose weight” [2, p. 245]. Thus, the distinction between healthy versus and unhealthy dieting is not always clearly defined in research and in our personal choices.

Although many studies have examined the beneficial role of religiosity in various health domains, the effects of religious engagement on disordered eating are rarely examined [3]. Despite the lack of studies on the relation between religiosity and disordered eating, religious practices often require a change in eating habits at some point or throughout the year. For instance, the Greek Orthodox Christian church follows the Nativity Fast, Lent, and the Assumption, and Muslims practice the Ramadan fasting [4]. Although fasting serves a religious purpose for some people, for others, this purpose has been used to mask religious fasting as a technique for weight control [5].

Intermittent fasting is a recent diet fad promoting weight lost through very low-caloric regimes spread on an alternating eating schedule [6]. Results from studies regarding whether fasting is a healthy or unhealthy weight loss/control behaviour are mixed. Some research suggests that fasting reduces body fat and mass, which in turn reduces risk of medical conditions, such as myocardial infarction [7] and type 2 diabetes [8]. On the other hand, periods of fasting are associated with poor mood, fatigue, and dizziness, and can be lethal in certain populations such as the elderly, patients with reactive hypoglycemia, children, pregnant individuals, and people engaged in strenuous physical work [9, 10]. The dangerous implications of extreme restriction of calorie intake cause researchers to be wary about recommending this diet for weight loss/control [10].

### The effect of religiosity on disordered eating

A systematic review [3] identified several benefits and risks of various aspects of religiosity related to disordered eating attitudes and behaviours. The main outcomes were that authentic religious beliefs and a profound relationship with one’s God were related to healthier eating habits and better body image, whereas shallow religious beliefs and an insecure attachment with one’s God were associated with greater disordered eating including body dissatisfaction [11, 12]. An experimental study compared women’s levels of body esteem after reading religious, versus non-religious, affirmations about their bodies before and after exposure to photos portraying the thin ideal [13]. Compared to a control group, women in both groups had increased body esteem after reading the body-related affirmations even after exposure to an appearance-related pressure. The difference between the religious and non-religious affirmation groups was not statistically significant. In their systematic review, Akrawi et al. [3] examined 15 articles and reported that six articles indicated positive relations between religiosity or spiritual and disordered eating, four reported negative relations, two displayed both directions, and three reported no significant relation. In sum, the role of religiosity in disordered eating and external influences remains unclear due to inconsistent and insufficient findings.

### The role of gender in religiosity and disordered eating

In the past, researchers reported that women had eating disorders at a rate that was 10 times greater than the rate reported by men [14]. Although more recent studies suggest that the disparity is only three times greater, there is a consensus that eating disorders occur more frequently in females. Further, internalization of appearance ideals differs between men and women; men experience appearance pressures stemming from peers, family, and the media to achieve the muscular ideal whereas women strive to attain the thin ideal [15].

There is limited literature on the manifestation of disordered eating attitudes and behaviours in males, especially when also considering the role of religiosity in which only seven studies were identified by Akrawi et al. [3]. The results of these studies showed a positive relation between aspects of religiosity and disordered eating [16], a negative relation [17–20], or an insignificant relation [21, 22]. None of the studies in this systematic review made direct gender comparisons according to religious status across disordered eating components. Considering the gender differences in patterns of disordered eating in men versus women, and the lack of research examining the role of religiosity in disordered eating research, the goals of the current study were to explore the interconnectedness of gender and religiosity on disordered eating attitudes and behaviours. The main objectives of this study were to: (a) corroborate and expand upon the implications of religiosity in perceiving family, peers, and the media pressures to achieve an ideal appearance; and (b) further our understanding on the interaction of gender and religiosity in disordered eating.

The first research objective examined whether levels of disordered eating and external influences varied according to religiosity. Although the influence of external pressures on disordered eating has been well-established in the literature, these influences have not been examined in relation to religiosity. Therefore, our hypotheses focused on the role of religiosity on appearance-related internalizations and pressures (i.e., family, peers, and the media) are exploratory.

The second research objective investigated if there were significant differences in patterns of disordered eating behaviours and attitudes of religious and non-religious men and women. We hypothesized that women would report greater levels of disordered eating attitudes and behaviours representing the thin ideal internalization such as Body Dissatisfaction, Cognitive Restraint, Purging, and Restricting. We also expected that men would indicate higher rates of disordered eating focused on muscular ideal internalization including Excessive Exercise, Binge Eating, Muscle Building. Negative Attitudes Toward Obesity should have proportionate rates between men and women since it strays away from both the thin and muscular ideals. Given that the differential effect of religiosity on gender has not yet been examined in disordered eating research, no hypothesis was formulated to predict this interaction.

## Method

### Participants

In total, 749 participants (ages 16–62 years) completed the online questionnaires, with 539 females (*M*_age_ = 27.98, *SD* = 10.94) and 209 males (*M*_age_ = 28.26, *SD* = 9.36). Too few participants identified as another gender identity (*n* = 10) to make meaningful, statistical comparisons. More of the participants identified as non-religious (57.68%; *n* = 432) versus 42.32% who identified as religious (*n* = 317).

### Measures

#### Demographic questionnaire

The demographic questionnaire (internally generated) included questions to collect information about age, gender, weight, height, and religiosity. The latter construct was assessed using the following: religious identity (i.e., “Do you identify with a religious affiliation?”), diet change (i.e., “In the past month, have you participated in a religious practice that required you to change your eating habits? If yes, please specify what change(s) were made”), and dieting purposes (i.e., “Was the change(s) in eating habits also used as a method of weight control in addition to religious purposes?”). To further investigate the potential differences among religious individuals, participants who answered “yes” to the latter question were defined as *high-risk* to disordered eating, whereas those who answered “no” were defined as *low-risk*.

#### Eating pathology symptoms inventory

The Eating Pathology Symptoms Inventory (EPSI) measures disordered eating attitudes and behaviours within the past month [25]. The EPSI is divided into eight subscales: Body Dissatisfaction (e.g., “I did not like how clothes fit the shape of my body;” α = 0.87), Binge Eating (e.g., “I ate until I was uncomfortably full;” α = 0.88), Cognitive Restraint (e.g., “I counted the calories of foods I ate;” α = 0.72), Purging (e.g., “I used diet pills;” α = 0.89), Restricting (e.g., “I skipped two meals in a row;” α = 0.86), Excessive Exercise (e.g., “I exercised to the point of exhaustion;” α = 0.86), Negative Attitudes Toward Obesity (e.g., “I felt that overweight people are lazy;” α = 0.88), and Muscle Building (e.g., “I thought my muscles were too small;” α = 0.79). The 45 items are answered using a 5-point Likert scale ranging from 0 (*never*) to 4 (*very often*). The EPSI has good psychometric qualities in non-clinical samples of both men and women [26].

#### Sociocultural Attitudes Toward Appearance Questionnaire-4

The Sociocultural Attitudes Toward Appearance Questionnaire-4 (SATAQ-4) assesses perceived societal pressures to achieve an ideal appearance [27]. The 22 items are divided into five subscales. Internalization–Thin/Low Body Fat (α = 0.84) assesses the preoccupations underlying the thin ideal internalization (e.g., “I want my body to look very thin”). Internalization–Muscular/Athletic (α = 0.90) examines the activities underlying the muscle ideal internalization (e.g., “It is important for me to look athletic”). The remaining three subscales include items referring to influences that may pressure the individual to achieve an ideal appearance: Family (α = 0.89), Peers (α = 0.92), and Media (α = 0.94). Examples of items in these subscales include “I feel pressure from family members to improve my appearance,” “My peers encourage me to get thinner,” and “I feel pressure from the media to look in better shape.” The items in the SATAQ-4 are answered on a five-point Likert scale ranging from 1 (*definitely disagree*) to 5 (*definitely agree*).

### Procedure

This study was approved by the University of New Brunswick  Research Ethics Board (REB File #056-2020). Participants were recruited from social media via Facebook (www.facebook.com; *n* = 249) and from SONA (*n* = 500), an online tool facilitating research participation for students enrolled in psychology courses at the University of New Brunswick. Social media participants had the opportunity to enter a draw prize for one of six $25 Amazon gift cards. University of New Brunswick student participants received bonus points applicable to a list of selected psychology courses of their choice. All participants complete the questionnaires through Qualtrics (www.qualtrics.com).

## Results

Prior to data analyses, we examined assumptions underlying the statistical analyses. The assumptions underlying independent samples *t* test, analysis of variance (ANOVA), and multivariate analysis of variance (MANOVA) were tested. The assumption of homogeneity of variance was examined using Levene’s test of homogeneity, and Welch’s *F* was used for ANOVAs, which adjusts the *F* ratio and degrees of freedom to reduce the probability of making a Type I error. Homogeneity of covariance was confirmed using the Box’s Test and although MANOVA is robust when group sizes are greater than 30, Pillai’s Trace was used. The assumption of normality was evaluated based on the skewness and kurtosis levels, which indicated a normal distribution within the absolute values of 1.5 and 3.0 respectively. The assumption of linearity was assessed with scatterplots (dependent variable vs. predicted values), which illustrated a linear relationship. Absence of multicollinearity between the dependent variables was indicated as the correlations were below 0.80. We identified multivariate outliers using Mahalanobis distance and removed from further analyses.

An examination of religiosity of the participants indicated that a proportionate number of males identified as religious (*n* = 105) and non-religious (*n* = 102), whereas more women identified as non-religious (*n* = 323) than religious (*n* = 209). Among the 317 participants who identified as religious, 12.30% reported that their religious practice required a change in their eating habits in the past month, and 10.68% reported that this change in eating habit was not only for religious purposes but also as a method of weight loss/control.

Table 1 contains direct quotes from participants explaining their change in eating habit, which categorized according to four main themes: vegetarianism/pescetarianism (*n* = 12), fasting (*n* = 7), dietary restraint (*n* = 2), and non-specific (*n* = 2). In fasting, participants indicated outright skipping a meal, whereas in dietary restraint, respondents reported lower calorie intake without skipping a meal. A relatively proportionate number of males and females reported these changes in their eating habits.

**Table 1:** Qualitative table incorporating religious participants’ response to their change(s) in eating habits

Change in eating habit	Quote from participant	Gender
Vegetarianism/pescetarianism	According to Islam, Muslims ban pigs, horses, donkeys, mules, dogs, and all animals that die of their own accord	M
	Don’t eat meat	F
	I didn’t eat any meat on Ash Wednesday	F
	Lent. No meat products	F
	Lent. Vegetarian for 40 days	F
	On Good Friday I could not eat meat, only fish, vegetables, grains, etc. [*sic*]	M
	Sometimes, the Catholic Church will suggest not to eat meat on Fridays as a sacrifice, but nothing too serious	M
	Vegetarian for lent	F
	Yeah Ramadan	M
	Yes, during Easter, I could not eat meat for the Holy Thursday and Good Friday	F
	Yes, stop taking pork	M
	Yes, I am fasting from meat on Fridays	F
Fasting	Fasting on a certain Saturday [*sic*]	F
	Fasting. No meat. 2 small meals plus one normal meal during the same day	F
	I didn’t eat breakfast	M
	My church does a “21 Day Fast” but encourages members to “fast” in aspects other than food-like 21 days social media-free, etc.	F
	Yes. I fast once a month	M
Dietary restraint	Lent, no desserts	F
	Lent [*sic*] fasting (junk food)	F
Non-specific	The food is light	F
	One is that all clean food given by God is to be eaten	M

*M* male, *F* female

A series of one-way ANOVAs compared reported rates of disordered eating and external pressures between non-religious participants (*n* = 437), respondents who answered “yes” in response to the question focused on the changes in eating habit associated with religiosity as a weight control behaviour (high-risk religious; *n* = 80), and those who answered “no” (low-risk religious; *n* = 232). The high-risk religious sample indicated significantly higher levels of Purging, Restricting, Excessive Exercise, Negative Attitudes Toward Obesity, Binge Eating, and Muscle Building than the non-religious and the low-risk religious sample. Further, high-risk respondents also had higher levels of Pressure from Family and Peers, and Internalization of the Muscular/Athletic ideal (see Table 2).

**Table 2:** Comparison of means of non-religious, low-risk and high-risk participants on disordered eating and environmental variables

Variable	Non-religious		Religious					
			Low-risk		High-risk			
	*M*	*SD*	*M*	*SD*	*M*	*SD*	*F*	*p*
Age	27.33	9.53	29.63	13.07	27.48	6.05	2.92	.056
BMI	25.99_a_	7.35	25.74_a_	6.21	22.16_b_	7.29	8.65	< .001
Disordered eating total	58.73_a_	24.72	59.80_a_	26.70	83.54_b_	28.71	26.32	< .001
Body dissatisfaction	14.20	6.97	13.68	6.54	14.16	5.35	0.47	.628
Cognitive restraint	5.44	3.11	5.68	2.79	6.13	2.43	2.52	.083
Purging	2.63_a_	4.24	3.10_a_	5.11	9.03_b_	6.30	37.95	< .001
Restricting	7.39_a_	5.58	7.74_a_	5.32	11.63_b_	4.13	32.79	< .001
Excessive exercise	7.27_a_	5.28	7.77_a_	4.81	10.23_b_	3.74	18.35	< .001
Negative attitudes obesity	5.40_a_	4.68	5.82_a_	4.70	9.65_b_	4.38	31.43	< .001
Binge eating	12.57_a_	7.03	11.70_a_	6.48	15.30_b_	5.87	10.60	< .001
Muscle building	3.81_a_	3.74	4.35_a_	4.31	8.13_b_	4.36	33.99	< .001
Pressure–family	9.40_a_	4.64	9.70_a_	4.75	12.83_b_	2.95	39.25	< .001
Pressure–peers	8.00_a_	4.17	8.07_a_	4.23	12.43_b_	3.42	55.76	< .001
Pressure–media	13.48_a_	5.38	13.41_a_	5.27	12.38_a_	3.46	3.04	.049
Internalization thin/low body fat	15.97	5.05	15.79	4.89	16.05	3.40	0.17	.847
Internalization muscular/athletic	13.84_a_	5.37	13.57_a_	5.36	16.16_b_	3.46	15.27	< .001

Means with different subscripts have statistically significant mean differences at the *p* = .05 level by Bonferroni’s post hoc test. Welch’s *F* was used for the *F* value

To address our second research question, a two-way MANOVA was used to determine whether there were significant differences between gender and religiosity across the disordered eating subscales. There was a statistically significant interaction effect between gender and religiosity, as well as significant main effects of gender and religiosity (see Table 3). The significant interaction, *F*(8, 720) = 4.43, *p* < 0.001, Pillai’s Trace = 0.047, η_p_^2^ = 0.05, indicated that the effect of religiosity on overall disordered eating differed for men and women. The interaction term and the effect of religiosity on disordered eating had small effect sizes following Cohen’s guidelines of small (η_p_^2^ = 0.01), medium (η_p_^2^ = 0.06), and large (η_p_^2^ = 0.14) partial eta squared effect sizes [28].

**Table 3:** Multivariate analysis of variance between gender and religiosity across disordered eating components

	Pillai’s trace	*F* (df)	*p*	η_p_^2^
*Overall Manova*				
Gender	.253	30.51 (8, 720)	< .001	.253
Religion	.052	4.93 (8, 720)	< .001	.052
Gender × religion	.047	4.43 (8, 720)	< .001	.047

EPSI subscale	Religiosity					Gender		Religiosity		Interaction	
		Yes		No							
		*M*	*SD*	*M*	*SD*	*F*	η_p_^2^	*F*	η_p_^2^	*F*	η_p_^2^
*Univariate main effects and interactions*											
Body dissatisfaction	Male	12.59	5.60	9.39	6.11	60.64	.077	2.80	.004	19.59***	.026
	Female	14.36	6.42	15.80	6.56						
Cognitive restraint	Male	5.85	2.52	5.00	2.81	1.11	.002	4.04*	.006	2.31	.003
	Female	5.75	2.84	5.62	3.12						
Purging	Male	7.44	6.54	3.26	5.06	38.76	.052	35.86	.046	19.63***	.026
	Female	3.10	5.04	2.51	4.00						
Restricting	Male	10.15	4.74	7.09	4.96	3.97	.005	13.49	.018	9.78**	.013
	Female	7.84	5.33	7.60	5.81						
Excessive exercise	Male	9.73	4.21	8.07	5.23	13.62***	.018	7.01**	.010	1.90	.003
	Female	7.64	4.82	7.12	4.82						
Neg. attitudes obesity	Male	8.55	4.34	6.77	4.87	32.64***	.043	10.72**	.015	1.71	.002
	Female	5.83	4.88	5.07	4.57						
Binge eating	Male	13.91	6.24	10.47	6.42	0.46	.001	3.80	.005	17.85***	.024
	Female	11.94	6.46	13.21	7.12						
Muscle building	Male	7.61	4.73	5.71	4.35	85.46***	.105	16.62	.022	3.17	.004
	Female	4.02	4.05	3.27	3.38						

*p < .05; **p < .01; ***p < .001.

**Fig. 1:**
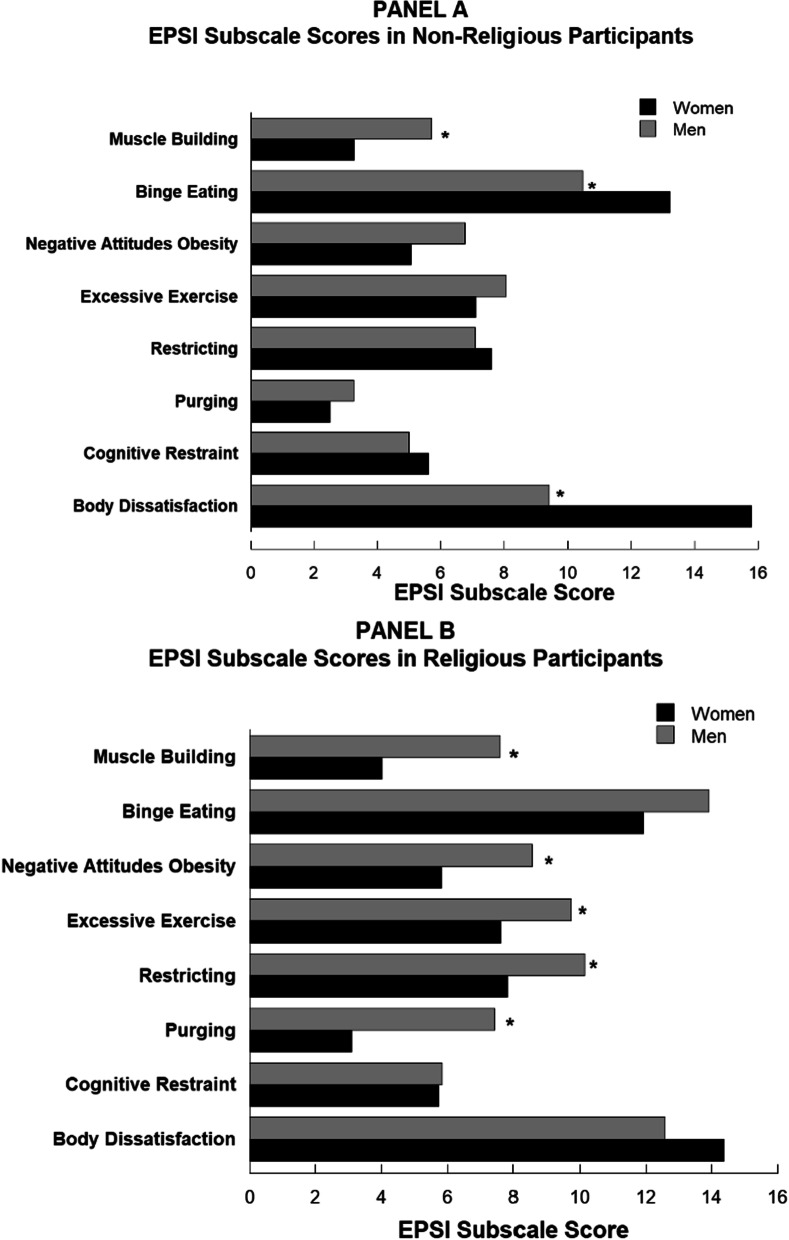
Eating Pathology Symptoms Inventory (EPSI) subscale scores for males and females, as a function of religious status

Overall, Fig. 1 shows that non-religious females reported significantly higher levels of Body Dissatisfaction and Binging than non-religious males. Conversely, non-religious males reported higher levels of Muscle Building than non-religious females. Among religious participants, males and females had similar levels of Body Dissatisfaction, Cognitive Restraint, and Binge Eating. Interestingly, religious males had higher levels of Purging, Restricting, Muscle Building and Negative Attitudes towards Obesity than religious females

**Fig. 2:**
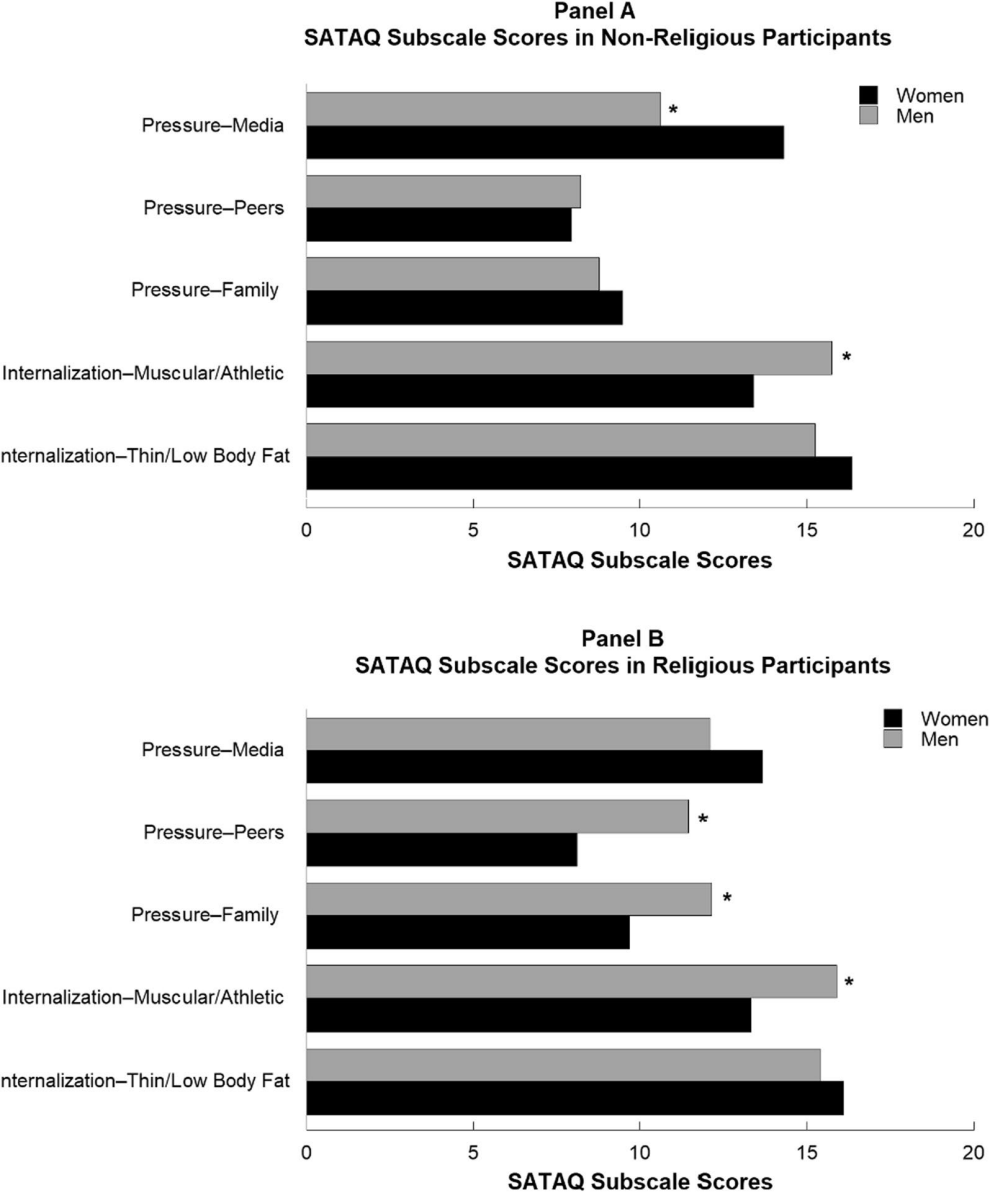
Sociocultural Attitudes Towards Appearance Questionnaire-4 (SATAQ-4) subscale scores for males and females, as a function of religious status

Further, to examine the effects of the external pressures and internalizations of sociocultural ideals a second two-way MANOVA was conducted, with SATAQ-4 subscale scores as the dependent variable. There was a small, but statistically significant interaction between gender and religiosity on the combined SATAQ-4 subscales, as well as significant main effects according to gender and religiosity (see Table 4). The significant interaction, *F*(5, 718) = 5.03, *p* < 0.001, Pillai’s Trace = 0.034, η_p_^2^ = 0.03, indicated that the effect of religiosity on appearance-related pressures and internalizations differed for men and women. Figure 2 illustrates that among non-religious participants, females reported significantly higher scores on Pressure from Media than males. Among religious participants, males reported higher scores on Pressure from Family and Peers.

**Table 4:** Multivariate analysis of variance between gender and religiosity across sociocultural attitudes towards appearance

	Pillai’s trace	*F* (df)	*p*	η_p_^2^
*Overall Manova*				
Gender	.180	31.47 (5, 718)	< .001	.180
Religion	.041	6.19 (5, 718)	< .001	.041
Gender × religion	.034	5.03 (5, 718)	< .001	.034

SATAQ subscale	Religious affiliation					Gender		Religious		Interaction	
		Yes		No							
		*M*	*SD*	*M*	*SD*	*F*	η_p_^2^	*F*	η_p_^2^	*F*	η_p_^2^
*Univariate main effects and interactions*											
Internalization: thin ideal	Male	15.37	3.27	15.19	4.40	5.78*	.008	< 0.001	< 0.001	.205	< .001
	Female	16.14	5.09	16.32	5.07						
Internalization: muscular ideal	Male	15.88	4.09	15.65	4.57	34.00***	.044	.048	.00	.015	< .001
	Female	13.36	5.36	13.32	5.35						
Pressure from family	Male	12.11	3.75	8.73	4.40	5.14	.007	22.19	.03	18.02***	.024
	Female	9.65	4.71	9.48	4.67						
Pressure from peers	Male	11.46	4.01	8.23	4.24	28.48	.038	23.41	.031	19.78***	.027
	Female	8.05	4.25	7.92	4.17						
Pressure from media	Male	12.05	4.84	10.64	4.85	40.69	.053	1.00	.001	5.58*	.008
	Female	13.73	5.22	14.30	5.25						

*p < .05; **p < .01; ***p < .001

## Discussion

Religiosity plays a beneficial role in several health domains, yet the research examining its implications in disordered eating is sparse [3]. The importance of further understanding the influence of religiosity on disordered eating is demonstrated by both inconsistent findings and insufficient research in the current literature [3]. We compared gender differences in religious versus non-religious individuals across several disordered eating attitudes and behaviours. Overall, the significant interactions between gender and religiosity suggests an interdependent effect of these variables on some components of disordered eating and internalization of societal appearance ideals. Specifically, the effect of religiosity in men versus in women was different in terms of body dissatisfaction, purging, restricting, and binge eating. Religious men reported higher levels of muscle building, negative attitudes towards obesity, excessive exercise, restricting, and purging than religious women. Among non-religious participants, results largely replicated previous results and indicated that, compared to men, women had higher levels of body dissatisfaction and binge eating as well as lower levels of muscle building.

Consistent with previous research [3], the current findings suggest that religiosity can have positive and negative relations with disordered eating attitudes and behaviours. It is possible that religious messages encouraging starvation and abominating gluttony may lead to increased levels of disordered eating [27]; our findings suggest that men may be more susceptible in engaging in disordered eating in order to follow religious rules than women. Considering that, to date, no study has examined the interaction of gender and religiosity across disordered eating components, more research is needed to further explore these conclusions. Future research should further explore gender differences across types and specific aspects of religiosity such as motivation to practice.

### The effects of religiosity

Religiosity is related to disordered eating attitudes and behaviours regardless of gender. Individuals who reported both types of changes in eating habits driven by religiosity, vegetarianism/pescetarianism versus fasting, had similar levels of disordered eating. A narrative review revealed that religious fasting has several empirically supported health benefits in terms of body weight and glycemia, cardiometabolic risk markers, and oxidative stress parameters [28]. Nonetheless, the number and severity of potential consequences associated with fasting should be considered when choosing a method for weight loss/control [10].

Individuals who reported using changes in eating habit for religious purposes as well as a method of weight control were at greater risk for engaging in disordered eating attitudes and behaviours, including purging, restricting, excessive exercise, negative attitudes toward obesity, binge eating, and muscle building, compared to non-religious and those who indicated not using religious fasting to lose/control weight. These findings support that, by its very nature, religiosity is not a risk factor of disordered eating. Instead, a subsample of religious individuals who are not completely genuine in their underlying intentions when engaging in religious fasting were more likely to engage in disordered eating [5].

### Gender differences in disordered eating

As expected, non-religious women experienced higher levels of body dissatisfaction than non-religious men. Interestingly, among religious participants, there were no differences in body dissatisfaction of men and women. Interestingly, this effect was driven by the fact that religious males had significantly greater body dissatisfaction than non-religious males. Thus, although body dissatisfaction was high for all female participants, the effects of religious affiliation in male participants warrants further examination. Further, the current results support previous research and indicate that women internalize the thin ideal more than men, regardless of their religiosity. This is likely due to a combination of factors, including the depiction of women in the media and lower levels of self-esteem consistently reported by women [29, 30]. In support of this, overall results indicated that women, regardless of religious affiliation, reported experiencing greater pressure from the media to attain a thin figure. Although both men and women are exposed to media pressures influencing their idealized body types, females are generally more affected as they are constantly given several indications about objectification in the Western culture [31].

As expected, compared to women, both religious and non-religious men indicated greater internalization of the muscular ideal, including excessive exercise and muscle building as well as higher levels of negative attitudes toward obesity. Some studies suggest that women’s appraisal of having a higher BMI is more negative than it is for males [32]; however, when other factors are considered, such as experiencing substance use [33], associated disordered eating behaviours [34], and overall poor health [35], men also report significant levels of weight stigmatization.

Unexpectedly, in the current study, among religious participants, men reported higher levels of purging and restricting than females. Although some researchers have begun considering the implication of gender in disordered eating during COVID-19 [36], direct gender comparisons of the prevalence of distinct disordered eating components have not been made. One study found that, compared to men, more women reported dieting prior to COVID-19 as well as increased emotional eating, food intake, and eating in response to negative affect during COVID-19 [37]. Thus, it is possible that the effects of COVID-19 were even more nuanced and differentially affected religious and non-religious individuals. In females, disordered eating attitudes and behaviours were high prior to the pandemic and remained high during the pandemic. Given that public religious activities were restricted, religious males may have turned to changes in eating (i.e., religious fasting) to participate in their religion. Future research could explore the interactive effects of gender and religiosity during health crises.

### Differences in sociocultural expectations

This study explored a novel research direction and examined the differential effect of religiosity by gender across external pressures and internalized sociocultural ideals. Overall, for both women and men, there were no statistically significant differences in internalization of the thin or muscular ideal as a function of religiosity. Interestingly, although religious and non-religious women reported similar pressures from family, peers, and the media to attain a body aligned with societal expectations, religious men experienced greater pressure from their family and peers than non-religious men. Considering these significant findings, future replication studies should address the gaps in literature regarding the differential effect of religiosity in males and females in constructs related to disordered eating (i.e., external pressures and internalized sociocultural ideals).

Studies have shown that peers and family are important influences regarding a person’s relation with religion [38]. Additionally, from a very young age, family members can influence their kins disordered eating through their own diet habits [39] and body dissatisfaction [40], and peers can influence disordered eating through discussions about aesthetic ideals [41] and social comparison. There is limited literature on the influence of family and peers on disordered eating in males and gender comparisons are quite rare. The current findings suggest family and peers can have an adverse effect on disordered eating, especially in religious men; however, more research should be conducted to future elucidate this relation.

Taken together, the effect of religiosity differs for adult men and women. Researchers have indicated that religiosity is associated with positive health outcomes when a person considers themselves religious; however, when an individual identifies as non-religious, religiosity is associated with poor health outcomes [42]. The current results suggest that, for males, religiosity may be associated with behaviours associated with disordered eating.

### Limitations

The data was collected during the COVID-19 pandemic (January–April 2020), a period that has significantly impacted the general population’s eating habits [37]. The non-probability, cross-sectional sampling does not allow inferences about causality. Given that our sample was not randomly selected, our results may not generalise to the broader population. Considering that this study did not address this, future research is needed to understand the driving factors underlying the gender differences in the relation between religiosity and disordered eating, such as motivation for faith.

## Conclusion

Religiosity can be beneficial in reducing disordered eating attitudes and behaviours, but, for males, it may also pose a risk. The current results indicated that for females who reported a religious affiliation had similar levels of disordered eating and sociocultural pressures as those who were non-religious. Interestingly, compared to non-religious males, males who identified as religious reported higher disordered eating behaviours and increased pressure from family, friends, and the media to maintain societal expectations of the ideal body. This study provides further information on the role of religiosity and religious fasting in disordered eating between men and women. The results strongly endorse the fact that future studies should continue exploring gender differences in the relation between religiosity and disordered eating.

## Data Availability

Supporting data is available from the corresponding author.
